# Effect of *GnRHR*, *BMP6* and *FSHR* Gene Pyramiding on Litter Traits of Goats

**DOI:** 10.3390/ani15101358

**Published:** 2025-05-08

**Authors:** Xinyue Yang, Yaokun Li, Baoli Sun, Yongqing Guo, Ming Deng, Dewu Liu, Guangbin Liu

**Affiliations:** 1 College of Animal Science, South China Agricultural University, Guangzhou 510642, China; xinyueyang1124@163.com (X.Y.); ykli@scau.edu.cn (Y.L.); baolisun@scau.edu.cn (B.S.); yongqing@scau.edu.cn (Y.G.); dengming@scau.edu.cn (M.D.); 2 State Key Laboratory of Animal Nutrition and Feeding, College of Animal Science and Technology, China Agricultural University, Beijing 100193, China

**Keywords:** *GnRHR*, *BMP6*, *FSHR*, litter size, pyramiding effect

## Abstract

Many studies have shown that only a few breeds of goat have the trait of high fertility, and genes play an important role in regulating litter size. This study investigated the effects of *GnRHR*, *BMP6*, and *FSHR* gene polymorphisms on litter traits in four goat breeds (Chongqing black, Chuanzhong black, Leizhou, and Nubian). Single nucleotide polymorphisms (SNPs) (g.75G > A in *GnRHR*, g.951T > C in *BMP6*, and g.-112C > T/g.3236C > A in *FSHR*) were genotyped using PCR-RFLP/HRM in 959 goats. Association analysis revealed significant correlations between these SNPs and litter size in the Chongqing, Chuanzhong, and Leizhou breeds, with genotypes AA (*GnRHR*), CC (*BMP6*), TT (*FSHR*), and AA (*FSHR*) linked to higher prolificacy. Polygene polymerizing effect analysis identified the optimal combinations (e.g., AATTCC, AACCAACC) with enhanced litter sizes. Tissue-specific qPCR in Chuanzhong goats showed *GnRHR*, *BMP6*, and *FSHR* were significantly more highly expressed in reproductive tissues (pituitary, breast, ovary, oviduct) of the prolific group than those in the non-prolific group. These SNPs serve as potential molecular markers for improving goat litter traits through polygenic selection, emphasizing the synergistic impact of multi-gene interactions on prolificacy.

## Simple Summary

With the increasing demand for goat meat by humans, increasing goat production to meet the demand for a healthy diet is an urgent task in the current livestock industry. In the production of small ruminants, it is particularly important to enhance their reproductive capacity, especially the litter-giving characteristics of does. Identifying key fecundity genes and utilizing polygene polymerizing effect analysis in goats can enhance litter size and improve production efficiency in the livestock industry. This study aimed to identify SNPs in the *GnRHR*, *BMP6*, and *FSHR* genes across four goat breeds, examine their association with litter size, and analyze the polygenic pyramiding effect on goat prolificacy. Our research identified four SNPs, and polygene polymerizing effect analysis further determined the optimal combination genotype that affects high prolificacy in goats. Overall, our results provide a foundation for molecular marker selection related to goat litter-size traits and a useful strategy for the further development of goat breeding.

## 1. Introduction

Utilizing molecular genetics techniques is a powerful approach for selecting and breeding animals with superior reproductive traits [1,2]. Numerous single nucleotide polymorphisms (SNPs) affecting goat litter size have been identified [3,4], yet most studies focus on single molecular markers, with limited research on multi-gene polymerization. Some traits or functions of biological organisms are controlled by multiple genes and belong to a regulatory network consisting of multiple genes and their products. Therefore, the effect of single genetic markers is relatively small and often cannot be practically applied in production, and polygenic polymerization is the first step to open the regulatory network of complex traits. In this study, the gonadotropin-releasing hormone receptor (*GnRHR*), bone morphogenetic protein 6 (*BMP6*) and follicle-stimulating hormone (*FSH*) receptor (*FSHR*) genes, which were the most prevalent, were used as candidate genes.

*GnRHR* gene is a high-affinity G-protein-coupled receptor of the pituitary gonadotropin membrane, which is located on chromosome 6 of sheep and is composed of 3 exons and 2 introns [5,6]. *GnRHR* gene as an essential mediator in the Gonadotropin-releasing hormone (*GnRH*) signaling pathway, *GnRHR* coupled to *GnRH*, plays an important role in activating the downstream pathway after stimulating a series of cascades to regulate reproduction [7]. In addition, *GnRHR* can transduce the signal of *GnRH* produced in the hypothalamus, regulate the synthesis and release of *FSH* and LH in the anterior pituitary, thereby promoting the growth, maturation and regulation of the reproductive performance of the gonad tissue.

*BMP6* gene is a member of the bone morphogenetic proteins (BMPs), which are members of the *TGF-β* superfamily. *BMP6* cDNA was first isolated from mouse embryos by Lyons et al. [8]. Recent studies have found that the *BMP6* gene directly participates in the regulation of the synthesis and secretion of reproduction-related hormones by inhibiting or promoting the activity of related enzymes or hormones in the body, thus affecting the formation and development of reproductive organs. For example, the *BMP6* gene can stimulate the synthesis of *FSH* via the autocrine or paracrine pathway [9,10,11]. El-Halawany et al. investigated the effect of *BMP6* gene polymorphisms on Egyptian sheep fecundity and its transcript expression in ovarian cells, but the result showed that the mutations of *BMP6* had no significance for litter size [12].

The *FSHR* gene, which contains 10 exons and 9 introns, is a glycoprotein family member of the G-protein-coupled receptor super-family, which mainly encodes the transmembrane domain and the intracellular domain in the 10th exon [13]. *FSHR* cDNA was first cloned from rat Sertoli cells in 1990 and its genetic mutations may affect the ability of *FSH* signal transduction [14]. More importantly, *FSHR* plays an important role in regulating the growth, development, differentiation and maturation of animal follicles. Borgbo et al. found that *FSHR* deficiency in Homo sapiens can lead to primary infertility [15]. Several studies have demonstrated that mutations of the *FSHR* gene were significantly associated with litter size in Hu sheep, Small Tailed Han sheep and Jining Grey goats [16,17,18]. Additionally, one study showed that the ovine *FSHR* gene was expressed widely and was significantly higher in sexual glands (testicle and ovary) than in other tissues [19].

This study analyzed four goat breeds: Chongqing black goat (CQ), Chuanzhong black goat (CZ), Leizhou goat (LZ), and Nubian goat (NS). Among them, NS were imported from abroad and then cultivated by local people, while the other three breeds are local goats in China. *GnRHR*, *BMP6* and *FSHR* genes have been extensively studied in pigs, sheep and other livestock, but most of them are based on the effects of a single-marker on fecundity. So far, no reports have been published on the analysis of the pyramiding effect of these three genes in goats. Although the polygene breeding of plants has achieved initial success, there were almost no reports of success on goat breeding. The aim of this study is to detect the SNPs of *GnRHR*, *BMP6*, and *FSHR* genes in four goat breeds, investigate their relationship with litter size, and analyze the polygene pyramiding effect on goat prolificacy, providing a basis for molecular marker selection for goat litter-size traits.

## 2. Materials and Methods

### 2.1. Experimental Materials

A total of 959 does were collected, including 290 LZ from Wuzhou company Shengzhou breeding goat farm (Wuzhou, China), 391 NS from Lezhu breeding goat farm of Xinxing County WENS Xinwang Goat Industry Co., Ltd. (Yunfu, China), 91 CQ and 187 CZ from Liangdong breeding goat farm of Xinxing County WENS Xinwang Goat Industry Co., Ltd. (Yunfu, China). All goats were raised in the same managed conditions. Venous jugular blood samples (5 mL per doe) were collected from these does in 2017, using acid citrate dextrose as an anticoagulant, and stored at −20 °C until DNA extraction; at the same time, the data on litter size in the first, second, third, or fourth parity of each individual were recorded in detail.

In addition, 8 CZ does were selected from Liangdong breeding goat farm of Xinxing County WENS Xinwang Goat Industry Co., Ltd. (Yunfu, China), which were equally divided into a non-prolific group (single lambs in three to four consecutive parities) and a prolific group (multiple lambs in three to four consecutive parities). All goats, aged 3–4 years, healthy, were in the artificial insemination system. Twelve tissue samples (heart, liver, spleen, lung, kidney, *longissimus dorsi muscle*, hypothalamus, pituitary, ovary, oviduct, uterus and breast) were taken from different parts of each goat, and immediately placed in liquid nitrogen and then stored at −80 °C until RNA extraction.

### 2.2. DNA Extraction

Genomic DNA was extracted from whole blood with Tissue DNA Kit D3396 kit, and detected by electrophoresis on 1% agarose gels (TaKaRa, Osaka, Japan); then, the concentration and purity of DNA fragment was detected by nanodrop spectrophotometer, and kept at −20 °C.

### 2.3. RNA Extraction and cDNA Synthesis

Total RNA was extracted from caprine tissues using a kit according to the instructions of the manufacturer (Total RNA Kit R6731, OMEGA, Bienne, Switzerland). RNA integrity was ascertained by agarose gel electrophoresis, and the purity and concentration of RNA were determined by spectrophotometer. A measure of 1 μg of total RNA was used for reverse transcription in a final volume of 20 μL according to the manufacturer’s recommendations (PrimeScript^TM^ RT reagent Kit with gDNA Eraser, TaKaRa, Japan). Synthesized cDNA was stored at −20 °C until further use.

### 2.4. Primers Design

Using Primer Primier 5.0 software, design universal primers and real-time PCR primers for *GnRHR*, *BMP6*, *FSHR*, and *β-actin* genes based on GenBank sequences (the accession number of goat genomic *GnRHR*, *BMP6* and *FSHR* are NC_019463.2, NC_019477.2, and NC_019460.2, respectively) were obtained and synthesized by Beijing Huada gene Biotechnology Co., Ltd. (Beijing, China). All primer information is listed in Table 1.

### 2.5. Genotyping of SNPs

PCR was carried out in a 10 µL reaction mixture containing 5 µL Taq DNA polymerase (Takara), 3.4 µL of ddH_2_O, 1 µL of goat genomic DNA, and 0.3 µL each of forward and reverse primer, respectively. The PCR amplification procedure was as follows: pre-denaturation at 94 °C for 2 min; followed by 35 cycles of denaturation at 94 °C for 30 s, annealing at 60 °C for 30 s, and extension at 72 °C for 30 s; extension at 72 °C for 5 min; and with a final preservation at 4 °C for 10 min. The PCR products of *GnRHR* and *BMP6* genes were digested separately with MspI (Thermo, Waltham, MA, USA) and HhaI (Thermo, Waltham, MA, USA) at 37 °C for 30 min in a 15 µL reaction mixture. The digestion system was as follows: 1 µL 10 × buffer, 8.5 µL of ddH_2_O, 0.5 µL restriction endonuclease (10 U/µL), and 5 µL PCR product. Then, the resultant fragments were separated by electrophoresis on 3% agarose gels.

HRM of *FSHR* gene was carried out in 10 µL reaction mixture containing 5 µL EvaGreen^®^ dye (Takara), 3.4 µL RNase-free water, 1 µL of goat genomic DNA, and 0.3 µL upstream and downstream primers, respectively. The HRM procedure was as follows: pre-denaturation at 94 °C for 2 min; followed by 40 cycles of denaturation at 94 °C for 10 s, annealing at 60 °C for 10 s, and extension at 72 °C for 5 s; finally, the fluorescence changes were monitored continuously during the procedure at 95 °C for 15 s, 55 °C 15 s, 55–95 °C (0.1 °C/s temperature rise rate), and the melting curve was obtained; the whole process takes about 10 min.

### 2.6. Reaction System and Conditions for qPCR

The cDNA of all tissue samples was used for qPCR analysis. qPCR amplification was performed in 20 µL of reaction mixture containing 10 µL of SYBR^®^ Green qPCR Master Mix (2×), 0.4 µL of ROX Reference Dye II (50×), 0.4 µL each of forward and reverse primer, 2 µL of cDNA, and 6.8 µL of ddH_2_O. PCR amplification was performed in triplicate wells, using the following procedure: 95 °C for 10 min, followed by 40 cycles of 95 °C for 15 s, and 60 °C for 10 s. Then, the melting curve was obtained, and the fluorescence changes were monitored continuously at 58 °C. The results were analyzed with the 2^−ΔΔCt^ method, using *β-actin* as the reference gene. The primers are listed in Table 1.

### 2.7. Statistical Analysis

Statistical analysis was conducted using GraphPad Prism version 8.0 (GraphPad Software, San Diego, CA, USA), with data presented as means ± SEM. Significant differences between two groups were determined using an unpaired two-tailed Student’s *t*-test or the Mann–Whitney U test for non-normally distributed samples. For data sets involving more than two groups, one-way ANOVA followed by the least significant difference (LSD) test or the non-parametric Kruskal–Wallis test was used, with SPSS 24.0 (SPSS Inc., Chicago, IL, USA). A *p*-value of less than 0.05 was considered statistically significant.

## 3. Results

### 3.1. Detection and Genotyping of Polymorphic Loci of GnRHR, BMP6 and FSHR Genes

**Figure 1:**
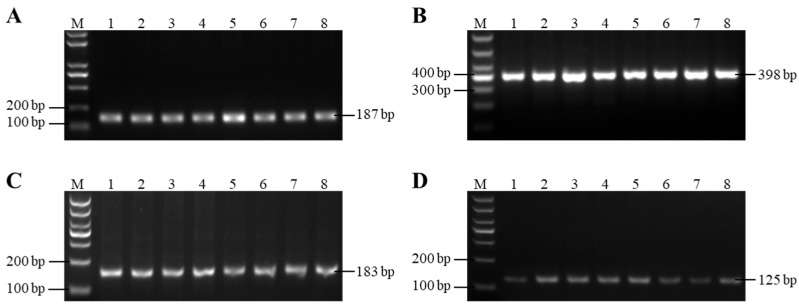
PCR amplification results of primers: (**A**) primer *GnRHR*, (**B**) primer *BMP6*, (**C**) primer *FSHR*-T1, (**D**) primer *FSHR*-T2. The numbers 1 to 8 denote the amplified fragment; M means DNA molecular weight marker (DL1000).

**Figure 2:**
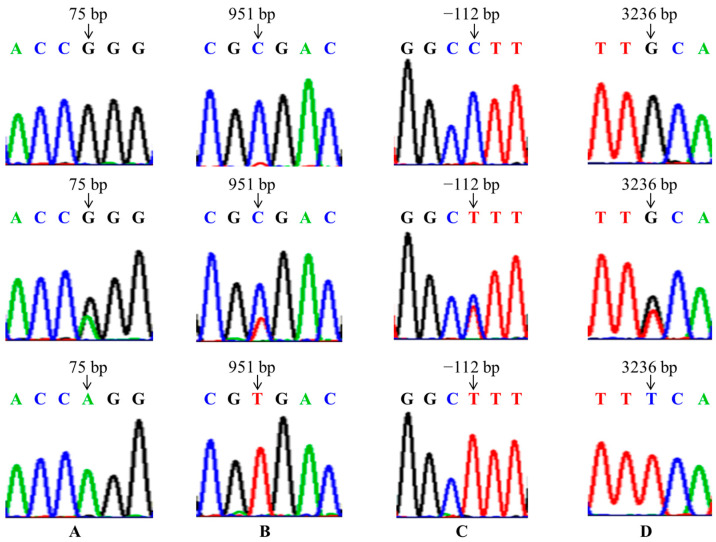
The sequencing map of polymorphic loci: (**A**) g.75G > A mutation of *GnRHR* gene, (**B**) g.951T > C mutation of *BMP6* gene, (**C**) the g.-112C > T mutation of *FSHR* gene, (**D**) the g.3236C > A mutation of FSHR gene.

Four pairs of primers of *GnRHR*, *BMP6* and *FSHR* genes were amplified, and detected by electrophoresis on 1.5% agarose gels. The length of the PCR amplified fragment was consistent with the target fragment, with clear bands and no stray bands, and could be directly used for sequencing (Figure 1). Then, the sequences were stitched and contrasted with gene reference sequences published in GeneBank. Four SNPs were identified in these three genes, namely g.75G > A locus, g.951T > C locus, g.-112C > T locus and g.3236C > A locus (Figure 2). Among them, g.75G > A locus is located in exon 1 of the *GnRHR* gene, and g.951T > C locus is located in exon 3 of the *BMP6* gene, but these two loci did not cause amino acid changes and belong to synonymous mutations; The g.-112C > T and g.3236C > A loci are located in the 5′URT and intron 10 of the *FSHR* gene, respectively.

**Figure 3:**
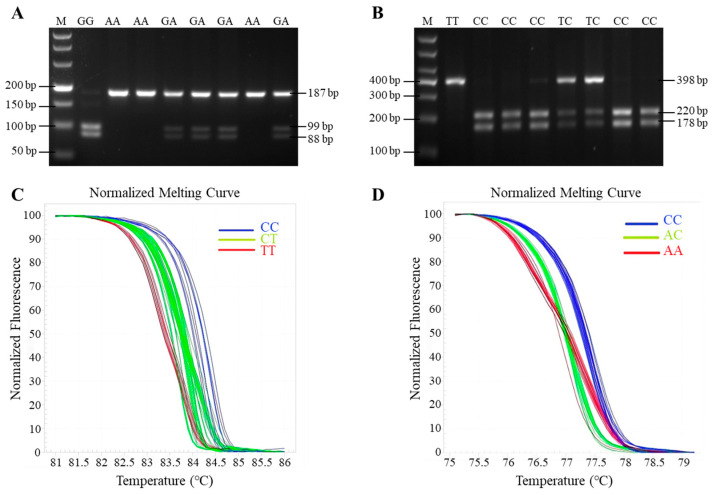
The results of genotyping: (**A**) the PCR-MspI-RFLP electrophoresis results of the g.75G > A mutation of *GnRHR* gene, (**B**) the PCR-HhaI-RFLP electrophoresis results of the g.75G > A mutation of *BMP6* gene, the genotypes are marked on the top of the lanes. M means DNA marker (DL2000). (**C**) PCR-HRM analysis of the g.-112C > T mutation of *FSHR* gene, (**D**) PCR-HRM analysis of the g.3236C > A mutation of *FSHR* gene.

After the g.75G > A locus of *GnRHR* gene was digested by MspI, there were three genotypes of GG (99 bp/98 bp), GA (187 bp/99 bp/98 bp), and AA (187 bp) in these four goat breeds (Figure 3A). The g.951T > C locus of *BMP6* gene was digested by HhaI, there were three genotypes of TT (398 bp), TC (398 bp/220 bp/178 bp), and CC (220 bp/178 bp) in these four goat breeds (Figure 3B).The g.-112C > T locus has three genotypes of CC, CT and TT (Figure 3C), also the g.3236C > A locus also has three genotypes of CC, AC and AA (Figure 3D) by the PCR-HRM analysis.

### 3.2. Genetic Parameter Analysis of SNPs

Heterozygosity (*He*), homozygosity (*Ho*), polymorphic information content (*PIC*) and effective allele numbers (*Ne*) are important parameters by which to judge the genetic variation in a population, and the different genetic parameters represent the essential genetic differences between groups. The genetic parameters of the g.75G > A locus of the *GnRHR* gene in LZ, CZ, NS and CQ are shown in Table 2. The *PIC* of these four goat breeds was between 0.25 and 0.5, so all of them show moderate polymorphism; the *He* and *Ne* of NS are lower, so the *Ho* is higher, indicating that the genetic performance of NS is stable, and it is easy to breed for conservation and raise the heterosis during breeding work; of the other three goat breeds, the *Ho* and *He* are both nearly 0.5, and the number of alleles detected and *He* are almost the same, indicating that the two alleles were evenly distributed among these three populations. The results of chi-square test (*χ*^2^) show that the Hardy–Weinberg equilibrium (HWE) bias occurred in the NS, indicating that the NS is in a non-natural equilibrium state, which may be a result of people’s long-term selection of a certain trait associated with the g.75G > A locus. The genotype distribution of the LZ, CZ and CQ is in the HWE state, which indicates that the g.75G > A locus of *GnRHR* gene might not be affected by current breeding measures, and their genetic changes are still random in the breeding process.

The genetic parameters of the g.951T > C locus of *BMP6* gene showed these four goat breeds were *PIC* < 0.25, so they all showed low polymorphism. The chi-square test showed that the g.951T > C locus was in the HWE state in the four goat breeds (Table 3).

According to Table 4 and Table 5, the *PIC* of g.-112C > T and g.3236C > A loci of the *FSHR* gene in the four goat breeds was between 0.25 and 0.5, so they all show moderate polymorphism. The chi-square test showed that the genotype distribution of these two loci had HWE bias in LZ, NS and CZ, but it was in the state of HWE in CQ.

### 3.3. Single Marker–Trait Association

Table 6, Table 7, Table 8 and Table 9 present the association analysis between four SNPs and litter size among individuals with different genotypes in CQ, CZ, LZ, and NS. The results indicate that there are no significant correlations between these four SNPs and litter size in NS. The association analysis result between the g.75G > A locus of *GnRHR* gene and litter size indicated that the does with genotype *AA* had 2.36 and 2.04 at the third birth and average litter size, and gave birth to more lambs than those with genotype *GA* and *GG* in CQ. The does with genotype *AA* were 0.69 and 0.28 more than the *GG* genotype ones at the second birth, respectively, with an average litter size of CZ. The does carrying genotype *AA* had more lambs when compared with the does carrying genotypes *GA* and *GG* in LZ.

The association analysis of the g.951T > C locus of the *BMP6* gene and litter size showed that does with the CC genotype had 2.28 and at their third birth and overall litter size of 1.98 lambs on average, respectively, compared to those with the TT genotype in CQ. In CZ, the does with the CC genotype produced significantly more lambs than those with the TT genotype at both the fourth birth and in overall litter size. In LZ, does with the CC and CT genotypes had more lambs than those with the TT genotype at their first, second births, and overall larger litter sizes. Additionally, the average litter size of does with the CC genotype was 0.45 more than those with the TT genotype.

The association analysis of the g.-112C > T locus of the *FSHR* gene and litter size revealed that does with the TT genotype had litter sizes of 3.00, 2.55, and 2.35 for their third, fourth births, and average litter size, respectively. These values were significantly higher than those with the CT and CC genotypes in CQ. Similarly, does with the TT genotype had significantly higher litter sizes compared to those with the CT and CC genotypes in CZ. However, there was no significant difference in kidding traits among the three genotypes in LZ.

The results from the association analysis between the g.3236C > A locus of *FSHR* gene and litter size showed that the litter size of genotype AA was significantly higher than those of genotypes CC and CA in the first birth and average litter size of CZ and LZ, but there was no significant difference between the litter sizes of the different genotypes in CQ.

### 3.4. Genetic Analysis of Polygene Pyramiding

The polygene pyramiding effect analysis of the three genes (*GnRHR*, *FSHR* and *BMP6*) on litter size is presented in Table 10, Table 11 and Table 12. The combination genotype AATTCC and AACCAACC had a significantly greater contribution on litter size than any other combination genotypes in CQ and CZ, respectively. The litter sizes of combination genotypes AAAACC and AACCCC were significantly higher than those in other combination genotypes in LZ. All of these four combination genotypes can be regarded as the optimal combination genotypes, where the combination genotypes AATTCC, AACCAACC and AAAACC were the combined forms of the dominant genotypes of SNPs. Interestingly, we found that the pyramiding effect of the three genes on litter size was far higher than those with either mutation alone.

### 3.5. Tissue Expression Analysis of Genes

**Figure 4:**
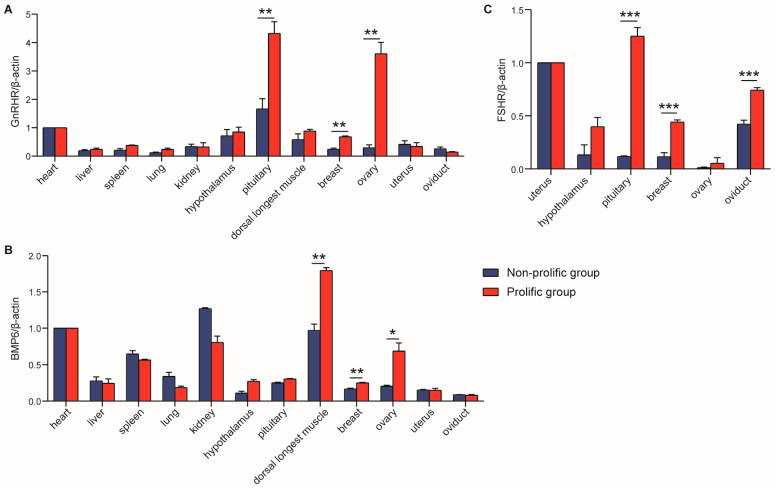
The mRNA expression level of *GnRHR* (**A**), *BMP6* (**B**) and *FSHR* (**C**) in different tissues of CZ. * *p* < 0.05, ** *p* < 0.01, *** *p* < 0.001.

The mRNA expression of *GnRHR* and *BMP6* genes was detected in 12 tissues including heart, liver, spleen, lung, kidney, *longissimus dorsi muscle*, hypothalamus, pituitary, ovary, oviduct, uterus and breast of CZ (Figure 4A,B), while the *FSHR* gene was expressed only in 6 tissues related to reproduction, such as hypothalamus, pituitary, ovary, oviduct, uterus and breast (Figure 4C). The expression of *GnRHR* gene in the pituitary, breast, and ovary of the prolific group was significantly higher than that of the non-prolific group, among which the expression of *GnRHR* in the pituitary was the highest (Figure 4A). The expression of the *BMP6* gene was highest in the *longissimus dorsi muscle*, and was higher in the prolific group than in the non-prolific group. Moreover, the *BMP6* expression in the breast and ovary tissues in the prolific group was significantly higher than in the non-prolific group (Figure 4B). In addition, the expression level of the *FSHR* gene in the pituitary, breast, and oviduct tissues in the prolific group was significantly higher than in the non-prolific group (Figure 4C). These results indicated that *GnRHR*, *BMP6*, and *FSHR* are closely related to the kidding performance of goats.

## 4. Discussion

In the present study, we selected the caprine *GnRHR*, *BMP6* and *FSHR* genes as candidate genes to analyze the effect of single-marker and multi-marker aggregation on litter size. The *GnRHR* gene plays a vital role in the regulation of reproductive performance in goats. Several studies indicated that does carrying the mutation of the *GnRHR* gene had significantly higher litter sizes compared with wild-type individuals [20,21,22]. The caprine *BMP6* gene is crucial in promoting normal fertility and skeletal growth in mice [23,24,25]. In the control study of sheep and rats, it was found that *BMP6* inhibited the progesterone production of both ovarian granulosa cells and stimulated the proliferation or survival of granulosa cells in rats. In contrast, it had no effect on the proliferation of ovarian granulosa cells but could inhibit its differentiation [10]. In addition, *BMP6* can maintain a lower apoptotic rate in cumulus cells by forming a local concentration gradient [26]. Numerous reports have shown that the *FSHR* has a central role in animal follicular development and litter traits [27,28,29]. Guo et al. research showed that allele C of 5′ regulatory region of the *FSHR* gene was a potential marker for improving litter size in goats [17]. Additionally, the g.47C > T mutation was found in the 5′ flanking region of the *FSHR* gene in the Small Tailed Han sheep and Hu sheep, and was significantly associated with litter size [19]. Overall, all these three genes could be selected as candidate genes for improving litter-size traits in goat husbandry.

In the present study, the mutations of *GnRHR* (g.75G > A), *FSHR* (g.-112C > T, g.3236C > A) and *BMP6* (g.951T > C) were found in four goat breeds, and were significantly associated with litter size in the CZ, CQ and DS breeds, so we can select the dominant genotypes to improve the fecundity of these three goat breeds in breeding work. But these four SNPs had no significant association with litter size in the NS. This may be because the NS had just been introduced into the field from Yunnan, and the growth environment was quite different, so its reproductive performance was still very unstable. Nonetheless, it may also be due to the small sample size or the distribution of the samples not being wide enough. Therefore, in order to make the research results more universal, we can further expand the sample size and sample distribution range in the following study.

Interestingly, the expression of *BMP6* in ovarian cells suggests that *BMP6* would be the most likely ligand regulating follicular maturation and ovulation rate through BMPR-IB [12]. In addition, studies have shown that the expression level of *GnRHR* mRNA is highest in the pituitary tissues of rats, pigs, cattle and sheep [30], which was consistent with our study. In this study, *GnRHR* and *BMP6* were detected in all 12 tissues, which implied that they played a role in promoting differentiation in many body tissues. The highest expression of *GnRHR* and *BMP6* was in the pituitary and *longissimus dorsi muscle*, respectively. Moreover, the *GnRHR* mRNA expression level in breast and ovary in the prolific group was significantly higher than that in the non-prolific group; *BMP6* mRNA expression level in the *longissimus dorsi muscle*, breast and ovary in the prolific group was prominently higher than that in the non-prolific group. The results of Pan et al. showed that ovine *FSHR* was expressed widely in detected tissues and was significantly higher in sexual glands (testicle and ovary) than in other tissues (heart, liver, spleen, lung, kidney, rumen, duodenum, muscle, fat, hypothalamus and pituitary) [19]. However, in our study, *FSHR* was checked only in reproduction-related tissues, and the expression level in the prolific group was significantly higher than the non-prolific group in the pituitary. This evidence suggested that *FSHR* might play a different role in goats and sheep. To some extent, the expression of candidate genes in the ovary is positively correlated with the reproductive performance of mammals [31,32], and the function of the ovary is generally regulated by the pituitary gland. In this study, the high expression of *GnRHR* and *FSHR* in the pituitary of the prolific group may promote the development and maturation of the follicle; it was also explained by the fact that they could regulate the ovulation rate of the ovary by their expression level, and then participate in the mechanism of regulating litter size in animals [33]. All these results further confirmed that these three genes are associated with the litter-size traits of goats.

So far, some studies have reported the effect of polygene pyramiding on litter size in sheep. Hanrahan et al. found that the ewes with mutations in both GDF9and BMP15 had a greater ovulation rate than those with either mutation alone in Cambridge and Belclare Sheep [34]. In the research of Chu et al., the Small Tailed Han sheep carried genotype BB/G+ (BMPR-IB/BMP-15) showed a greater litter size than those with only one mutation [35]. The result of Wang et al. indicated that mutations among *BMPR-IB*, *BMP15* and *FSHR* genes were found and the association analysis revealed that sheep with multiple markers had a higher litter size than those with single marker or two markers, and the combination genotype BB/G+/CC of *BMPR-IB*, *BMP15* and *FSHR* genes was considered the superior genotype [18], which is consistent with the present study. There were four SNPs detected in the current experimental populations. Polygene polymerizing effect analysis revealed that the CQ and CZ with combination genotypes AATTCC and AACCAACC, and LZ with combination genotypes AAAACC and AACCCC had more kids than any other combination genotypes or those with only one predominant genotype among the does. Moreover, the combination genotypes AATTCC, AACCAACC and AAAACC were the combination of the predominant genotypes of *GnRHR*, *BMP6* and *FSHR* genes, indicating that polygene polymerizing may have a greater effect on contributing to the litter size of goats, and the combination genotypes AATTCC, AACCAACC, AAAACC and AACCCC could be considered as the superior genotypes and used as molecular markers in breeding work.

Obviously, it is unrealistic to predict that the does that are selected will have a high litter size in the future if they rely solely on molecular markers to detect the genotype of the base. There are many factors that affect the litter size of does, including temperature, nutrition, disease, and stress response. If the does carry a dominant genotype, it can only show that the does have the potential for high fecundity, and their potential is also influenced by external factors during the process of growth and development. If the environment is conducive to the growth and development of the does, the potential of their prolificacy is likely to be exerted, otherwise their potential will be buried. It can be seen that in order to give full play to the role of molecular markers related to litter size in production and breeding, we must pay attention to the feeding and management level of the goats at the same time, so as to get better breeding effects.

## 5. Conclusions

In summary, the polymerization effect of the *GnRHR*, *BMP6* and *FSHR* genes of goats were analyzed for the first time. The results revealed that the optimal combination genotypes (AATTCC, AACCAACC, AAAACC and AACCCC) were a polymerization of the dominant genotypes of single molecular markers, but the number of individuals with optimal combination genotypes was not enough in the experimental population; more samples should be selected to investigate the pyramiding effect of more genes in future studies, and to obtain effective molecular markers for polygene polymerization breeding of goats. In addition, the high expression of these three genes in the ovary or pituitary of the prolific group of goats further explained their correlation with litter traits.

## Figures and Tables

**Table 1:** Primer sequences of PCR-RFLP/PCR-HRM and real-time PCR.

	Gene Name		Primer Sequence (5′→3′)	Product Size (bp)	Annealing Temperature (°C)	Endonuclease/HRM
PCR-RFLP/PCR-HRM primer	*GnRHR*	F	TCTTGAAGCTGTATCAGCCATA	187	56	MspI
		R	GTGTTGAAAATTGTGGAGAGTAGA			
	*BMP6*	F	TTTTAGGGCTCCATGTACTCC	398	59	HhaI
		R	TTTCCTCTGGGTGCTGTG			
	*FSHR*-T1	F	TACCAGCTCCCAATGCAGAC	183	58	HRM
		R	GACAGAGTCGATGGTGGCAT			
	*FSHR*-T2	F	TACACACTCATCCCCCTA	125	60	HRM
		R	GTGCTCTGTCAGCTCTTG			
Real-time PCR primer	*GnRHR*	F	GGCTGAGGACCCTAAAGATG	134	60	-
		R	ACCATGTCAGGATCAAACCA			
	*BMP6*	F	CTTTCTCAACGACGCCGATAT	106	60	-
		R	TCACCGCCTCACCCTCAG			
	*FSHR*	F	CGAGTCATCCCAGAAGGAG	137	60	-
		R	GGCAGGTTGGAGAACACATT			
	*β-actin*	F	AGCCTTCCTTCCTGGGCATGGA	113	60	-
		R	GGACAGCACCGTGTTGGCGTAGA			

F: forward primer; R: reverse primer.

**Table 2:** The genetic parameters of *GnRHR* gene g.75G > A locus.

Breed	Number	Allelic Frequency		Genotypic Frequency (*N*)			*χ*^2^ (HWE)	*PIC*	*Ho*	*He*	*Ne*
		G	A	GG	GA	AA					
CQ	91	0.44	0.56	0.24 (22)	0.40 (36)	0.36 (33)	3.53	0.37	0.51	0.49	1.97
CZ	187	0.46	0.54	0.21 (40)	0.49 (92)	0.29 (55)	0.02	0.37	0.50	0.50	1.99
LZ	290	0.63	0.37	0.40 (115)	0.46 (134)	0.14 (41)	0.04	0.36	0.53	0.47	1.88
NS	391	0.25	0.75	0.05 (19)	0.40 (155)	0.55 (217)	180.91 **	0.30	0.63	0.37	1.59

Note: the numbers in the parentheses represent the number of the genotypes, *df* = 2, *χ*^2^_0.05_ = 5.99, *χ*^2^_0.01_ = 9.21; the superscript with ** means extremely significant difference (*p* < 0.01), no mark indicates that the difference is not significant (*p* > 0.05). 0.25 < *PIC* < 0.5 is moderately polymorphic.

**Table 3:** The genetic parameters of *BMP6* gene g.951T > C locus.

Breed	Number	Allelic Frequency		Genotypic Frequency (*N*)			*χ*^2^ (HWE)	*PIC*	*Ho*	*He*	*Ne*
		C	T	CC	CT	TT					
CQ	91	0.34	0.67	0.13 (12)	0.41 (37)	0.46 (42)	0.70	0.35	0.55	0.45	1.80
CZ	187	0.25	0.75	0.07 (13)	0.36 (68)	0.57 (106)	0.21	0.31	0.62	0.38	1.60
LZ	290	0.31	0.69	0.07 (19)	0.48 (140)	0.45 (131)	5.27	0.34	0.57	0.43	1.74
NS	391	0.34	0.66	0.12 (48)	0.44 (170)	0.44 (173)	0.39	0.35	0.55	0.45	1.81

Note: the numbers in the parentheses represent the number of the genotypes, *df* = 2, *χ*^2^_0.05_ = 5.99, *χ*^2^_0.01_ = 9.21. 0.25 < *PIC* < 0.5 is moderately polymorphic.

**Table 4:** The genetic parameters of *FSHR* gene g.-112C > T locus.

Breed	Number	Allelic Frequency		Genotypic Frequency (*N*)			*χ*^2^ (HWE)	*PIC*	*Ho*	*He*	*Ne*
		C	T	CC	CT	TT					
CQ	91	0.69	0.31	0.51 (46)	0.37 (34)	0.12 (11)	1.38	0.34	0.57	0.43	1.74
CZ	187	0.57	0.43	0.42 (78)	0.30 (57)	0.28 (52)	26.79 **	0.37	0.51	0.49	1.96
LZ	290	0.48	0.52	0.32 (93)	0.31 (90)	0.37 (107)	41.40 **	0.37	0.50	0.50	2.00
NS	391	0.46	0.54	0.29 (114)	0.33 (129)	0.38 (148)	43.90 **	0.37	0.50	0.50	1.99

Note: the numbers in the parentheses represent the number of the genotypes, *df* = 2, *χ*^2^_0.05_ = 5.99, *χ*^2^_0.01_ = 9.21; the superscript with ** means extremely significant difference (*p* < 0.01), no mark indicates that the difference is not significant (*p* > 0.05). 0.25 < *PIC* < 0.5 is moderately polymorphic.

**Table 5:** The genetic parameters of *FSHR* gene g.3236C > A locus.

Breed	Number	Allelic Frequency		Genotypic Frequency (*N*)			*χ*^2^ (HWE)	*PIC*	*Ho*	*He*	*Ne*
		C	A	CC	CA	AA					
CQ	91	0.58	0.42	0.37 (34)	0.41 (37)	0.22 (20)	2.54	0.37	0.51	0.49	1.95
CZ	187	0.48	0.52	0.35 (65)	0.26 (48)	0.40 (74)	44.07 **	0.37	0.50	0.50	2.00
LZ	290	0.40	0.598	0.23 (67)	0.34 (99)	0.43 (124)	24.35 **	0.37	0.52	0.48	1.93
NS	391	0.42	0.58	0.28 (111)	0.27 (105)	0.45 (175)	78.54 **	0.37	0.51	0.49	1.95

Note: the numbers in the parentheses represent the number of the genotypes, *df* = 2, *χ*^2^_0.05_ = 5.99, *χ*^2^_0.01_ = 9.21; the superscript with ** means extremely significant difference (*p* < 0.01), no mark indicates that the difference is not significant (*p* > 0.05). 0.25 < *PIC* < 0.5 is moderately polymorphic.

**Table 6:** Association analysis between g.75G > A locus of *GnRHR* gene and litter size of four goat breeds.

Breed	Genotype	Litter Size (*N*)				
		First Parity	Second Parity	Third Parity	Fourth Parity	Average
CQ	GG	1.50 ± 0.51 (22)	2.00 ± 0.56 (20)	1.74 ± 0.56 ^b^ (19)	2.00 ± 0.82 (13)	1.77 ± 0.30 ^b^ (22)
	GA	1.56 ± 0.50 (36)	1.82 ± 0.77 (33)	1.97 ± 0.68 ^b^ (29)	1.92 ± 0.64 (25)	1.80 ± 0.33 ^b^ (36)
	AA	1.82 ± 0.77 (33)	1.91 ± 0.59 (22)	2.36 ± 0.84 ^a^ (31)	2.04 ± 0.66 (26)	2.04 ± 0.47 ^a^ (33)
CZ	GG	1.70 ± 0.69 (40)	1.62 ± 0.49 ^c^ (37)	2.00 ± 0.84 (32)	2.04 ± 0.79 (25)	1.75 ± 0.48 ^b^ (40)
	GA	1.79 ± 0.67 (92)	1.93 ± 0.78 ^b^ (86)	2.08 ± 0.62 (77)	1.82 ± 0.61 (56)	1.90 ± 0.42 ^ab^ (92)
	AA	1.73 ± 0.62 (55)	2.31 ± 0.77 ^a^ (49)	2.02 ± 0.75 (42)	1.92 ± 0.69 (26)	2.03 ± 0.46 ^a^ (55)
LZ	GG	1.47 ± 0.50 ^b^ (115)	1.64 ± 0.52 (56)	1.33 ± 0.58 ^b^ (3)	—	1.52 ± 0.44 ^b^ (115)
	GA	1.46 ± 0.50 ^b^ (134)	1.75 ± 0.46 (81)	2.33 ± 0.58 ^a^ (3)	—	1.57 ± 0.42 ^b^ (134)
	AA	1.76 ± 0.62 ^a^ (41)	1.83 ± 0.64 (24)	2.00 ± 0.00 ^ab^ (4)	—	1.79 ± 0.51 ^a^ (41)
NS	GG	1.79 ± 0.54 (19)	—	—	—	—
	GA	1.87 ± 0.51 (155)	—	—	—	—
	AA	1.83 ± 0.53 (217)	—	—	—	—

Note: numbers in parentheses are numbers of individuals that belong to the respective genotypes. The different superscript lowercase letters in the same column represent significant level at *p* < 0.05, and same letter or no letter represents no significant difference (*p* > 0.05).

**Table 7:** Association analysis between g.951T > C locus of *BMP6* gene and litter size of four goat breeds.

Breed	Genotype	Litter Size (*N*)				
		First Parity	Second Parity	Third Parity	Fourth Parity	Average
CQ	TT	1.33 ± 0.49 (12)	1.60 ± 0.97 (10)	1.44 ± 0.73 ^b^ (9)	1.60 ± 0.55 (5)	1.39 ± 0.39 ^b^ (12)
	CT	1.65 ± 0.59 (37)	1.92 ± 0.55 (36)	1.97 ± 0.71 ^ab^ (31)	1.96 ± 0.59 (27)	1.92 ± 0.30 ^a^ (37)
	CC	1.71 ± 0.67 (42)	1.95 ± 0.65 (39)	2.28 ± 0.72 ^a^ (39)	2.06 ± 0.76 (32)	1.98 ± 0.38 ^a^ (42)
CZ	TT	1.77 ± 0.60 (13)	1.82 ± 0.98 (11)	1.78 ± 0.67 (9)	1.29 ± 0.49 ^b^ (7)	1.64 ± 0.47 ^b^ (13)
	CT	1.77 ± 0.72 (68)	1.85 ± 0.76 (65)	1.95 ± 0.68 (60)	1.90 ± 0.59 ^a^ (40)	1.87 ± 0.42 ^ab^ (68)
	CC	1.75 ± 0.63 (106)	2.07 ± 0.73 (96)	2.15 ± 0.72 (82)	1.97 ± 0.71 ^a^ (60)	1.96 ± 0.46 ^a^ (106)
LZ	TT	1.21 ± 0.42 ^b^ (19)	1.07 ± 0.27 ^b^ (14)	2.00 ± 0.00 (1)	—	1.20 ± 0.38 ^b^ (19)
	CT	1.46 ± 0.53 ^b^ (140)	1.73 ± 0.55 ^a^ (81)	1.83 ± 0.75 (6)	—	1.57 ± 0.44 ^a^ (140)
	CC	1.60 ± 0.52 ^a^ (131)	1.86 ± 0.39 ^a^ (66)	2.00 ± 0.00 (3)	—	1.65 ± 0.45 ^a^ (131)
NS	TT	1.90 ± 0.37 (48)	—	—	—	—
	CT	1.80 ± 0.54 (170)	—	—	—	—
	CC	1.87 ± 0.54 (173)	—	—	—	—

Note: numbers in parentheses are numbers of individuals that belong to the respective genotypes. The different superscript lowercase letters in the same column represent significant level at *p* < 0.05, and same letter or no letter represents no significant difference (*p* > 0.05).

**Table 8:** Association analysis between g.-112C > T locus of *FSHR* gene and litter size of four goat breeds.

Breed	Genotype	Litter Size (*N*)				
		First Parity	Second Parity	Third Parity	Fourth Parity	Average
CQ	CC	1.67 ± 0.60 (46)	1.86 ± 0.61 (42)	1.76 ± 0.55 ^c^ (37)	1.87 ± 0.50 ^b^ (31)	1.78 ± 0.36 ^b^ (46)
	CT	1.59 ± 0.61 (34)	1.84 ± 0.77 (32)	2.10 ± 0.79 ^b^ (31)	1.86 ± 0.71 ^b^ (22)	1.86 ± 0.37 ^b^ (34)
	TT	1.64 ± 0.81 (11)	2.18 ± 0.41 (11)	3.00 ± 0.447 ^a^ (11)	2.55 ± 0.82 ^a^ (11)	2.35 ± 0.34 ^a^ (11)
CZ	CC	1.65 ± 0.72 (78)	1.87 ± 0.68 (71)	1.97 ± 0.57 ^b^ (62)	1.89 ± 0.53 (45)	1.83 ± 0.51 ^b^ (78)
	CT	1.79 ± 0.59 (57)	1.96 ± 0.78 (53)	1.94 ± 1.68 ^b^ (46)	2.00 ± 0.78 (31)	1.91 ± 0.36 ^ab^ (57)
	TT	1.87 ± 0.63 (52)	2.13 ± 0.84 (48)	2.28 ± 0.85 ^a^ (43)	1.81 ± 0.75 (31)	2.02 ± 0.45 ^a^ (52)
LZ	CC	1.48 ± 0.50 (93)	1.68 ± 0.55 (53)	1.75 ± 0.50 (4)	—	1.55 ± 0.43 (93)
	CT	1.56 ± 0.52 (90)	1.77 ± 0.48 (44)	2.00 ± 0.00 (4)	—	1.64 ± 0.45 (90)
	TT	1.48 ± 0.56 (107)	1.73 ± 0.51 (64)	2.00 ± 1.41 (2)	—	1.56 ± 0.47 (107)
NS	CC	1.84 ± 0.54 (114)	—	—	—	—
	CT	1.83 ± 0.48 (129)	—	—	—	—
	TT	1.86 ± 0.54 (148)	—	—	—	—

Note: numbers in parentheses are numbers of individuals that belong to the respective genotypes. The different superscript lowercase letters in the same column represent significant level at *p* < 0.05, and same letter or no letter represents no significant difference (*p* > 0.05).

**Table 9:** Association analysis between g.3236C > A locus of *FSHR* gene and litter size of four goat breeds.

Breed	Genotype	Litter Size (*N*)				
		First Parity	Second Parity	Third Parity	Fourth Parity	Average
CQ	CC	1.62 ± 0.65 (34)	1.90 ± 0.80 (30)	2.00 ± 0.82 (28)	2.00 ± 0.67 (23)	1.82 ± 0.45 (34)
	CA	1.68 ± 0.67 (37)	1.94 ± 0.53 (36)	2.11 ± 0.68 (35)	1.96 ± 0.74 (28)	1.93 ± 0.34 (37)
	AA	1.60 ± 0.50 (20)	1.79 ± 0.63 (19)	2.06 ± 0.85 (16)	2.00 ± 0.58 (13)	1.88 ± 0.40 (20)
CZ	CC	1.51 ± 0.56 ^b^ (65)	1.94 ± 0.85 (62)	1.98 ± 0.71 (55)	2.17 ± 0.92 ^a^ (35)	1.85 ± 0.43 ^b^ (65)
	CA	1.69 ± 0.47 ^b^ (48)	2.02 ± 0.79 (41)	1.92 ± 0.67 (38)	1.70 ± 0.47 ^b^ (30)	1.83 ± 0.34 ^b^ (48)
	AA	2.01 ± 0.75 ^a^ (74)	1.97 ± 0.66 (69)	2.19 ± 0.71 (58)	1.81 ± 0.46 ^b^ (42)	2.00 ± 0.52 ^a^ (74)
LZ	CC	1.40 ± 0.52 ^b^ (67)	1.68 ± 0.53 (40)	1.50 ± 0.57 (2)	—	1.48 ± 0.47 ^b^ (67)
	CA	1.47 ± 0.52 ^ab^ (99)	1.82 ± 0.54 (60)	1.75 ± 0.50 (4)	—	1.60 ± 0.42 ^ab^ (99)
	AA	1.58 ± 0.52 ^a^ (124)	1.67 ± 0.47 (61)	2.25 ± 0.50 (4)	—	1.63 ± 0.46 ^a^ (124)
NS	CC	1.82 ± 0.59 (111)	—	—	—	—
	CA	1.83 ± 0.51 (105)	—	—	—	—
	AA	1.87 ± 0.48 (175)	—	—	—	—

Note: numbers in parentheses are numbers of individuals that belong to the respective genotypes. The different superscript lowercase letters in the same column represent significant level at *p* < 0.05, and same letter or no letter represents no significant difference (*p* > 0.05).

**Table 10:** Pyramiding effect of *GnRHR* (g.75G > A), *FSHR* (g.-112C > T) and *BMP6* (g.951T > C) on litter size in CQ.

Loci	Combination Genotype	Litter Size (*N*)				
		First Parity (69)	Second Parity (61)	Third Parity (56)	Fourth Parity (36)	Average (69)
g.75G > A/g.-112C > T/g.951T > C	GGCCTC	1.43 ± 0.53 ^b^ (7)	2.14 ± 0.38 ^ab^ (7)	1.67 ± 0.52 ^bc^ (6)	2.00 ± 0.71 (5)	1.79 ± 0.16 ^bc^ (7)
	GACCCC	1.67 ± 0.50 (9)	2.00 ± 0.82 (7)	1.86 ± 0.38 ^bc^ (7)	1.80 ± 0.45 ^b^ (5)	1.77 ± 0.36 ^c^ (9)
	GACCTC	1.88 ± 0.35 (8)	1.63 ± 0.74 ^bc^ (8)	1.40 ± 0.55 ^c^ (5)	2.00 ± 0.00 (5)	1.82 ± 0.23 ^bc^ (8)
	GACTTC	1.40 ± 0.55 ^b^ (5)	1.80 ± 0.45 (5)	2.20 ± 0.45 ^ab^ (5)	2.00 ± 0.71 (5)	1.88 ± 0.11 ^bc^ (5)
	AACCTC	2.00 ± 0.89 (6)	2.17 ± 0.41 ^ab^ (6)	1.67 ± 0.52 ^bc^ (6)	1.83 ± 0.41 ^b^ (6)	2.07 ± 0.33 (6)
	AACCCC	2.00 ± 0.63 (6)	2.00 ± 0.00 (6)	2.33 ± 0.52 ^ab^ (6)	1.80 ± 0.45 ^b^ (5)	2.07 ± 0.27 (6)
	AATTCC	2.00 ± 1.00 (5)	2.40 ± 0.55 ^a^ (5)	2.80 ± 0.45 ^a^ (5)	2.60 ± 0.89 ^a^ (5)	2.42 ± 0.39 ^a^ (5)
	AACTCC	2.20 ± 0.84 ^a^ (6)	1.40 ± 0.55 ^c^ (5)	2.80 ± 1.30 ^a^ (5)	—	2.13 ± 0.48 ^ab^ (6)
	GGCTCC	1.50 ± 0.55 (6)	2.33 ± 0.52 ^a^ (6)	1.67 ± 0.52 ^bc^ (6)	—	1.92 ± 0.28 ^bc^ (6)
	AACTTC	1.33 ± 0.52 ^b^ (6)	1.83 ± 0.75 (6)	2.20 ± 0.45 ^ab^ (5)	—	1.82 ± 0.43 ^bc^ (6)
	GACTCC	1.40 ± 0.55 ^b^ (5)	—	—	—	1.75 ± 0.26 ^bc^ (5)

Note: numbers in parentheses are numbers of individuals that belong to the respective genotypes. The different superscript lowercase letters in the same column represent significant level at *p* < 0.05, and same letter or no letter represents no significant difference (*p* > 0.05). The number of combination genotypes less than 5 was not analyzed.

**Table 11:** Pyramiding effect of *GnRHR* (g.75G > A), *FSHR* (g.-112C > T, g.3236C > A) and *BMP6* (g.951T > C) on litter size in CZ.

Loci	Combination Genotype	Litter Size (*N*)				
		First Parity (75)	Second Parity (62)	Third Parity (41)	Fourth Parity (23)	Average (75)
g.75G > A/g.-112C > T/g.3236C > A/g.951T > C	GACTAACC	2.11 ± 0.33 ^a^ (9)	1.78 ± 0.44 (9)	2.00 ± 0.54 (8)	2.00 ± 0.00 ^a^ (5)	2.01 ± 0.30 ^b^ (9)
	GATTCACC	1.67 ± 0.52 (6)	1.83 ± 0.98 (6)	2.33 ± 1.03 (6)	1.33 ± 0.52 ^b^ (6)	1.78 ± 0.31 ^b^ (6)
	GACCAATC	2.17 ± 1.17 ^ac^ (6)	1.50 ± 0.55 (6)	2.00 ± 0.00 (5)	2.20 ± 0.45 ^a^ (5)	1.88 ± 0.59 ^b^ (6)
	GACCAACC	1.50 ± 0.54 ^bc^ (8)	2.00 ± 0.00 (7)	2.14 ± 0.69 (7)	1.57 ± 0.54 ^b^ (7)	1.76 ± 0.47 ^b^ (8)
	GATTAACC	2.33 ± 0.82 ^a^ (6)	2.17 ± 0.98 (6)	2.20 ± 0.45 (5)	—	2.08 ± 0.60 ^ab^ (6)
	AACCCCTC	1.40 ± 0.55 ^b^ (5)	2.00 ± 0.71 (5)	1.60 ± 0.55 (5)	—	1.65 ± 0.27 ^b^ (5)
	AACCCCCC	1.29 ± 0.49 ^b^ (7)	2.29 ± 0.95 (7)	2.20 ± 0.45 (5)	—	1.89 ± 0.48 ^b^ (7)
	GACTCACC	1.60 ± 0.55 (5)	2.20 ± 0.45 (5)	—	—	1.96 ± 0.09 ^b^ (5)
	GACTAATC	2.00 ± 0.71 (5)	1.80 ± 0.84 (5)	—	—	1.96 ± 0.25 ^b^ (5)
	GACTCCCC	1.86 ± 0.69 (7)	2.17 ± 1.17 (6)	—	—	2.06 ± 0.26 ^b^ (7)
	GACCCCCC	1.40 ± 0.55 ^bc^ (5)	—	—	—	1.60 ± 0.42 ^b^ (5)
	AACCAACC	2.33 ± 1.03 ^a^ (6)	—	—	—	2.56 ± 0.81 ^a^ (6)

Note: numbers in parentheses are numbers of individuals that belong to the respective genotypes. The different superscript lowercase letters in the same column represent significant level at *p* < 0.05, and same letter or no letter represents no significant difference (*p* > 0.05). The number of combination genotypes less than 5 was not analyzed.

**Table 12:** Pyramiding effect of *GnRHR* (g.75G > A), *FSHR* (g.3236C > A) and *BMP6* (g.951T > C) on litter size in LZ.

Loci	Combination Genotype	Litter Size (*N*)		
		First Parity (274)	Second Parity (142)	Average (274)
g.75G > A/g.3236C > A/g.951T > C	GGCCTC	1.19 ± 0.40 ^b^ (16)	1.56 ± 0.53 ^c^ (9)	1.33 ± 0.39 ^b^ (16)
	GGCCCC	1.42 ± 0.51 ^bc^ (12)	2.00 ± 0.00 ^ac^ (5)	1.56 ± 0.45 ^bc^ (12)
	GGCATC	1.47 ± 0.51 ^bc^ (19)	1.70 ± 0.67 (10)	1.59 ± 0.42 ^bc^ (19)
	GGCACC	1.47 ± 0.51 ^bc^ (19)	2.00 ± 0.00 ^a^ (10)	1.61 ± 0.43 ^bc^ (19)
	GGAATC	1.59 ± 0.50 (22)	1.40 ± 0.52 ^bc^ (10)	1.50 ± 0.48 b^c^ (22)
	GGAACC	1.74 ± 0.45 (19)	1.63 ± 0.52 (8)	1.66 ± 0.44(19)
	GACCTC	1.33 ± 0.49 ^b^ (15)	1.90 ± 0.57 ^ac^ (10)	1.50 ± 0.50 ^bc^ (15)
	GACCCC	1.67 ± 0.50 (9)	1.80 ± 0.45 (5)	1.61 ± 0.49 (9)
	GACATC	1.42 ± 0.51 ^bc^ (19)	1.79 ± 0.43 (14)	1.62 ± 0.33 ^bc^ (19)
	GACACC	1.45 ± 0.51 ^bc^ (20)	1.90 ± 0.32 ^ac^ (10)	1.55 ± 0.43 ^bc^ (20)
	GAAATC	1.48 ± 0.51 ^bc^ (33)	1.72 ± 0.46 (18)	1.62 ± 0.42 ^bc^ (33)
	GAAACC	1.50 ± 0.51 ^bc^ (28)	1.93 ± 0.26 ^ac^ (15)	1.63 ± 0.40 ^bc^ (28)
	AACATC	1.50 ± 0.76 (8)	2.00 ± 1.00 ^ac^ (5)	1.67 ± 0.54 (8)
	AACACC	1.78 ± 0.44 ^acd^ (9)	1.88 ± 0.64 ^ac^ (8)	1.80 ± 0.43 ^ac^ (9)
	AACCCC	1.86 ± 0.38 ^ac^ (7)	1.60 ± 0.55 (5)	1.79 ± 0.27 ^ac^ (7)
	AAAACC	2.00 ± 0.76 ^a^ (8)	—	2.00 ± 0.76 ^a^ (8)
	AAAATC	1.67 ± 0.52 (6)	—	1.78 ± 0.40 ^ac^ (6)
	GAAATT	1.60 ± 0.55 (5)	—	1.50 ± 0.50 ^bc^ (5)

Note: numbers in parentheses are numbers of individuals that belong to the respective genotypes. The different superscript lowercase letters in the same column represent significant level at *p* < 0.05, and same letter or no letter represents no significant difference (*p* > 0.05). The number of combination genotypes less than 5 was not analyzed.

## Data Availability

Data are contained within the article.

## Abbreviations

The following abbreviations are used in this manuscript:

*GnRHR*	Gonadotropin-releasing hormone receptor
*BMP6*	Bone morphogenetic protein 6
*FSHR*	Follicle-stimulating hormone receptor
*GnRH*	Gonadotropin-releasing hormone
CQ	Chongqing black goat
CZ	Chuanzhong black goat
LZ	Leizhou goat
NS	Nubian goat
*He*	Heterozygosity
*Ho*	Homozygosity
*PIC*	Polymorphic information content
*Ne*	Effective allele numbers
HWE	Hardy–Weinberg equilibrium
